# 798. Incomplete Contact Investigation and Risk of Developing Tuberculosis Among Healthcare Professionals After Tuberculosis Exposure

**DOI:** 10.1093/ofid/ofab466.994

**Published:** 2021-12-04

**Authors:** Thana Khawcharoenporn, Kongporn Noisang

**Affiliations:** Thammasat University, Bangkok, Krung Thep, Thailand

## Abstract

**Background:**

Tuberculosis (TB) contact investigation is recommended for healthcare professionals (HCPs) after TB exposure. However, association between no participation in or incomplete contact investigation and subsequent TB development has not been well-described. This study aims to determine TB incidences and factors associated with TB development among HCPs requiring contact investigations.

**Methods:**

We conducted a prospective cohort study among Thai HCPs with TB exposure from January 2013 to December 2017. Contact investigations, including baseline TB and latent tuberculosis infection (LTBI) screening and follow-up at 3 months after TB exposure, were recommended to all HCPs. The two-step tuberculin skin test (TST) was used for LTBI testing. All HCPs were followed for 2 years for TB development.

**Results:**

Of the 342 HCPs with TB exposure included in the study, 311 (91%) participated in the contact investigations and 252 (74%) completed baseline TB and LTBI screening. Among the 210 HCPs with negative baseline TST, 45 (21%) completed the follow-up tests. The overall incidence of TB was 2.92/100 person-years. HCPs who did not complete follow-up TST had significantly higher TB incidence than those completed baseline and follow-up TST (3.55 vs. 0/100 person-years; P=0.01). No participation in the contact investigation and no chest radiograph performed at baseline were the independent factors associated with TB development among the HCPs [adjusted odds ratio (aOR) 6.69; P< 0.001 and aOR 8.85; P=0.01, respectively]. Contact with an index patient with concomitant TB at extrapulmonary sites (aOR 49.76, 10.03-246.99; P< 0.001) and with negative sputum AFB but positive sputum GeneXpert MTB/RIF (aOR 3.18, 1.35-7.50; P=0.008) were independently associated with no participation in the contact investigation.

**Conclusion:**

The findings indicate the risk of TB development among the HCPs who did not undergo or complete contact investigations and underscore the need for interventions to improve contact investigation participation and completeness.

**Disclosures:**

**All Authors**: No reported disclosures

